# Analysis of Magnetic Nondestructive Measurement Methods for Determination of the Degradation of Reactor Pressure Vessel Steel

**DOI:** 10.3390/ma14185256

**Published:** 2021-09-13

**Authors:** Gábor Vértesy, Antal Gasparics, Ildikó Szenthe, Madalina Rabung, Melanie Kopp, James M. Griffin

**Affiliations:** 1 Centre for Energy Research, 1121 Budapest, Hungary; gasparics.antal@energia.mta.hu (A.G.); szenthe.ildiko@energia.mta.hu (I.S.); 2 Fraunhofer Institute for Nondestructive Testing (IZFP), 66123 Saarbrücken, Germany; Madalina.Rabung@izfp.fraunhofer.de (M.R.); Melanie.Kopp@izfp.fraunhofer.de (M.K.); 3 Future Transport and Cities Research Centre, Coventry University, Coventry CV1 5FB, UK; ac0393@coventry.ac.uk

**Keywords:** magnetic nondestructive evaluation, reactor pressure vessel, neutron irradiation embrittlement, magnetic adaptive testing, micromagnetic multiparameter microstructure and stress analysis 3MA, Barkhausen noise measurement, steel degradation, ductile to brittle transition temperature

## Abstract

Nondestructive magnetic measurement methods can be successfully applied to determine the embrittlement of nuclear pressure vessel steel caused by neutron irradiation. It was found in previous works that reasonable correlation could be obtained between the nondestructively measured magnetic parameters and destructively determined ductile-to-brittle transition temperature. However, a large scatter of the measurement points was detected even in the cases of the non-irradiated reference samples. The reason for their scattering was attributed to the local inhomogeneity of material. This conclusion is verified in the present work by applying three different magnetic methods on two sets of Charpy samples made of two different reactor steel materials. It was found that by an optimal magnetic pre-selection of samples, a good, linear correlation can be found between magnetic parameters as well as the ductile-to-brittle transition temperature with low scattering of points. This result shows that neutron irradiation embrittlement depends very much on the local material properties.

## 1. Introduction

Nuclear power plants (NPPs) have a key role within the energy production landscape. An extremely important aspect is their safety, so inspection of a power plant’s integrity is crucial, especially for the long-term operation. The most important part of the pressurized and boiling water reactors is the reactor pressure vessel (RPV). Their primary aging process is the irradiation generated material embrittlement and it is one of the most important lifetime limiting factors. This process, caused by the influence of the long-term and high-energy neutrons, generates changes in the mechanical properties [1], which are inspected periodically. However, the inspection of radiation embrittlement is not an easy task at all. So-called surveillance samples are put inside the vessel and after a certain period they are tested. Mechanical Charpy impact testing is the standard way of evaluation of the embrittlement [2]. The ductile-to-brittle transition temperature (DBTT) determined by Charpy impact testing is the authorized parameter that refers to embrittlement in the nuclear industry. However, this destructive measurement technique requires many samples, and the error of measurement is high. Concerted efforts have been made to continuously develop effective nondestructive methods for inspection of RPVs. Magnetic methods seem to be useful for this purpose since the reactor pressure vessel is made of ferromagnetic steels. A general overview can be found in Reference [3] about the application of nondestructive magnetic methods.

In several recent works, different nondestructive magnetic methods have been applied for detection of neutron irradiation generated embrittlement of nuclear reactor pressure vessel material. One of them is the so called magnetic adaptive testing, MAT based on the measurement of minor magnetic hysteresis loops [4,5]. Another one, the magnetic Barkhausen noise technique, is also suitable to detect the irradiation effects on RPV steel [6]. Finally, there is the 3MA method (micromagnetic multiparameter microstructure and stress analysis), which combines several different magnetic methods [7].

The general conclusion of these efforts was that a reasonable correlation had been found between the nondestructively measured magnetic parameters and the destructively measured DBTT if the above mentioned methods are applied [8,9,10]. It seems that magnetically measured parameters have a better potential to characterize the material embrittlement than the conventional destructive methods. However, the scatter of measurements points has been found to be rather large in all of these experiments.

The possible reason of this big scatter has been interpreted in a recent paper [11]. An important finding of this work is that the scatter of measurements points very probably can be explained by local material inhomogeneity. The embrittlement depends also very much on the initial material conditions. This fact is surprising, because the measured samples were prepared from the same RPV block, and from a predefined depth. The initial material conditions probably are connected with the microstructure of the samples, but the microstructure itself was not investigated; instead, we concentrated our attention to the interpretation of the magnetic measurements.

Considering the importance of these results for the future potential application of magnetic measurements and even for the whole nuclear industry, the results should be verified carefully. This is the purpose of the present work: Two series of standard Charpy samples made of two different types of RPV steel were measured both before and after neutron irradiation, and the three different magnetic measurement methods were applied systematically on the same specimens. The outcomes of these non-destructive methods have been evaluated jointly.

## 2. Materials and Methods

### 2.1. Materials and Mechanical Tests

For our investigations, two types of RPV materials were chosen, an Eastern RPV material (15Kh2NMFA) and a Western RPV material (A508 Cl.2). ISO-V Charpy samples were manufactured at SCK CEN [12,13] by cutting them out from ¾ depth in the case of A508 Cl.2 specimens, and from the ¼ depth in the case of 15Kh2NMFA specimens. According to the ASTM E23-16b standard the orientation of samples was selected as T-L.

The chemical composition of the samples are given in Table 1 and Table 2 for both materials. It was measured by a “Spectromax LMX06” Spark Atomic Emission Spectrometer (Ametek/Spectro [14]) according to the standard ASTM E415. Heat treatment of steel forgings means quenching and tempering including post-weld heat treatment.

**Figure 1:**
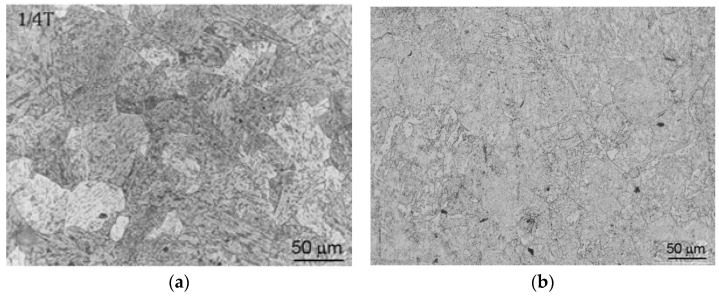
Optical microscopy performed in etched condition to observe the grain boundaries on an A508 CL.2 (**a**) and on an 15Kh2NMFA (**b**) sample.

The as-received samples’ microstructure of is a mixed tempered ferrite–bainite structure. As an illustration, typical microstructures of the two investigated materials, performed on non-irradiated samples are shown in Figure 1. After preparation of Charpy samples from Western and Eastern RPV material, one portion was mechanically tested and the other portion was nondestructively investigated. Following the magnetic measurements, these samples were divided into three sets for the neutron irradiation. E > 1 MeV neutron irradiation was performed in the primary water pool of the BR2 reactor at different irradiation levels with a fluence at a temperature between ~100–120 °C. Applied fluence levels were between 1.55 × 10^19^ n/cm^2^ and 7.90 × 10^19^ n/cm^2^.

Four Charpy samples for each irradiation condition were investigated. The irradiated samples were nondestructively tested. After that, destructive mechanical tests were performed. They were investigated by an instrumented pendulum (ISO 148-1 and ASTM E23) for the as-received non-irradiated and neutron irradiated materials.

The DBTT (i.e., its curve and the temperature where this curve bypasses the 41J criteria) can be determined by a series of Charpy impact tests carried out at different temperatures of the test set specimens. The transition temperature curve itself is determined by mathematical regression analysis, since a-priory unknown temperature value is to be derived which value becomes available just following the physical experiments. However, this statistical approach fades out the differences between the single samples of the test set and instead, provides a single DBTT value for the whole test specimen set. In addition, note the scattering of all measurements along the whole transition function to be fitted is relevant from a DBTT perspective, but only the measurement uncertainties of this transition curve around the point it by passes the 41J criteria or, where it has a slope. For instance, the scattering of the upper shelf energy (USE) is irrelevant in this case.

**Figure 2:**
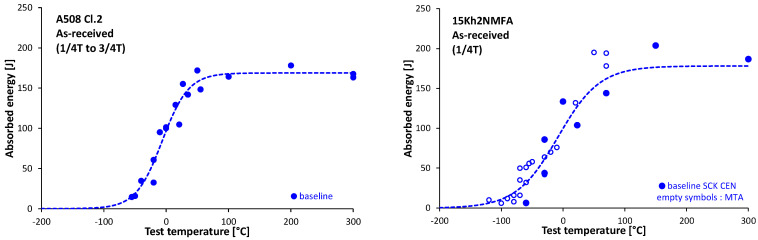
The scattering of the Charpy impact test measurements and the fitted ductile to brittle transition temperature curve that fades the specimens’ inhomogeneities in the case of A508 Cl.2, and of 15Kh2NMFA type material.

The scattering of the impact tests and the results of the regression analysis can be seen in Figure 2 in the case of A508 Cl.2 and 15Kh2NMFA type material. This figure also illustrates the preliminary assumption of this paper: comparing the outcomes of the individual non-destructive measurements to a statistical mean value obtained on non-ideal specimens will lead to scattering which cannot be attributed to the uncertainty of the non-destructive approach solely. This scattering can be seen in Figure 2, demonstrating the differences between the tested specimens. Therefore, these differences are related to the material inhomogeneities, and these are reflected in the mentioned NDE results as scattering. Correlation between transition temperature change and neutron fluence was found for both steels. Results are given in Table 3 and Table 4 for the A508 Cl.2 and for the 15kHNMFA steels, respectively.

Altogether 13 samples from 15Kh2NMFA material were measured before and after neutron irradiation, samples Nos. 166, 167, 168, 169, 171, 172, 173, 175, 176, 178, 181, 183, 185, and 11 samples from A508 Cl.2 material Nos. 572, 573, 575, 578, 579, 581, 583, 586, 587, 588, 591.

### 2.2. Magnetic Adaptive Testing

Magnetic adaptive testing (MAT) is a recently developed method of magnetic hysteresis measurement. The main point of this technique is that series of minor hysteresis loops are measured systematically, in contrast to the conventional hysteresis measurements, where major (saturation to saturation) hysteresis loops are recorded. The details of the measurement can be found in Reference [5]. As it was proven in many experiments, investigating several types of degradation of ferromagnetic materials, led to good correlation between the optimally chosen MAT descriptors and those parameters (usually determined destructively), characterize the actual material degradation. Sensitivity of MAT descriptors supersedes the sensitivity of conventional hysteresis measurements.

Samples are measured by a magnetizing yoke, attached directly to the sample surface. The size of the yoke fits the size of samples. Measurement starts with a careful demagnetization of samples by decreasing amplitude alternating magnetizing field. Samples then magnetized by a magnetizing current with a triangular waveform, starting from zero and increasing the amplitude step-by-step. Permeability loops are detected by a pick-up coil, wounded around a yoke leg. In the case of linearly increasing the magnetizing current, the pick-up coil’s output signal changes proportionally with the differential magnetic permeability of the whole magnetic circuit.

From points of the obtained minor permeability loops a permeability matrix is calculated and matrix elements are compared with the corresponding elements of the reference (in our case non irradiated) sample. From this, a big data pool is generated, and relevant parameters are chosen that characterize (with large sensitivity and simultaneously with good reproducibility) the modification of material properties due to different material degradation.

**Figure 3:**
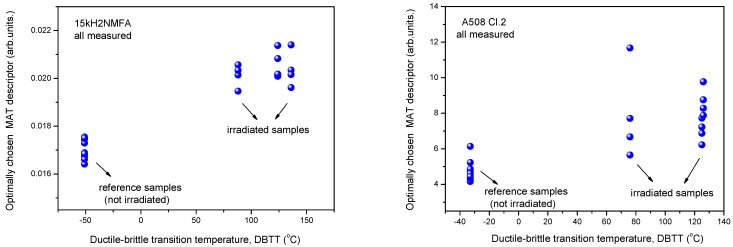
Optimally chosen MAT descriptor vs. transition temperature for all measured 15kH2NMFA and A508 Cl.2 samples.

**Figure 4:**
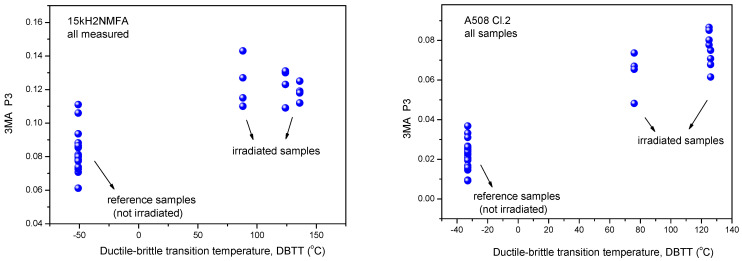
Dependence of the amplitude of third harmonics P3 parameter of 3MA as a function of transition temperature for all measured 15kH2NMFA and A508 Cl.2 samples.

**Figure 5:**
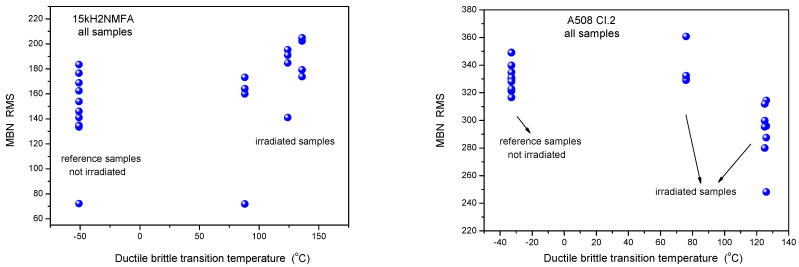
MBN RMS parameter as a function of transition temperature for all measured 15kH2NMFA and A508 Cl.2 samples.

As mentioned above, the first and most probable reason for the scatter of magnetic parameters vs. DBTT could be the error of magnetic measurement itself. In considering this, a careful analysis of MAT measurements was conducted. The result of this analysis is given in the Appendix of Reference [11]: The error of the total MAT evaluation has been found lower than 1% by taking into account of all possible uncertainties. This means that the error of MAT measurement and evaluation cannot be responsible for the big scatter of points, which can exceed in certain cases: 20%, as shown in Figure 3, Figure 4 and Figure 5. Similar conclusions can be made for the experimental error of 3MA and MNB measurements also.

### 2.3. Micromagnetic Multiparameter Microstructure and Stress Analysis

The Fraunhofer Institute for Nondestructive Testing developed the 3MA approach (3MA = micromagnetic multiparameter microstructure and stress analysis) which allows materials characterization to determine industry-relevant characteristics (hardening depth (CHD, SHD or NHD), hardness, yield and ultimate strength and DBTT. This method is suitable for measurements on active materials in hot cells. The measuring principle is rested on the correlation between the mechanical properties of ferromagnetic materials and their magnetic properties. This correlation is connected with the microstructure interaction with both the magnetic structure (consisting of magnetic domain separated by Bloch walls) as well as the dislocations [15,16].

The 3MA approach uses several parameters derived from three micromagnetic methods listed below [17]:

- Eddy currents (EC) are generated in the material under the influence of AC magnetic field. They depend on the σ electrical conductivity and on the µ magnetic permeability of the material, and they result a magnetic field with opposite direction to the originally applied magnetic field. It means, that σ and µ of the material has an influence on the excitation coil’s impedance. This impedance is measured.
- Analysis of incremental permeability (IP) is a method of separating the magnetic permeability information from the electrical conductivity information. For application of this method, the material should be magnetized with a low-frequency AC magnetic field and a continuous EC impedance analysis should be performed at a higher frequency. Considering the change of the coil impedance as a function of the magnetic field strength leads to an incremental permeability plot. In such a way, a qualitative correlation of the impedance change throughout the magnetic hysteresis and the magnetic field strength at maximum permeability (usually correlated with coercivity measured by means of magnetic hysteresis analysis) is obtained.
- Harmonics analysis (in time domain signal) of magnetizing current is used to describe the magnetic hysteresis behavior of the materials by applying one-sided access sensor. For this purpose, a magnetization electromagnet should be applied, which is driven by a sinusoidal voltage. A receiver coil measures the magnetizing current.

The impedance of the electromagnet coil changes as a consequence of the hysteretic correlation between the B magnetic flux density and H magnetizing field. In such a way, the current in the electromagnet contains harmonics, but it is not sinusoidal. The measured magnetizing current exhibits distortion due to the hysteresis in magnetic circuit. Fundamental and harmonic components can be numerically determined by a fast Fourier analysis, and thus distortions of the magnetizing current can be quantified. The harmonic components calculated by this procedure make possible the determination of the material properties.

These methods differ in terms of the analysis depth and mechanisms and deliver more than 20 parameters, which correlate qualitatively with material properties. Generally, 3MA systems are consisting of a probe, which contains a magnetization unit with a coil to capture the magnetic response of the material, a 3MA device for the excitation of the magnetization and preprocessing the measuring signals via a PC for measurement control and data processing. Different material depths and areas can be investigated depending on the properties of the magnetization unit as well on the parameters of the measurement. Micromagnetic methods can therefore analyze a controllable fraction of the sample volume.

The 3MA process should be calibrated on a calibration set of samples (with well-known properties, such as DBTT or hardness) [10,18]. For mechanical-technological materials characterization, the measuring parameters are registered by the PC and are further processed having performed all measurements and analyses, the software delivers the magnetic fingerprint (MFP) of the material properties, which can be used for quantitative and qualitative materials characterization. More than one measurement parameter is used proper for materials characterization. It is necessary to ensure increased robustness contra disturbing influences such as material variations and surface condition. For the calibration regression analyses, pattern recognition or other machine-learning algorithms can be used.

### 2.4. Barkhausen Noise Measurement

MBN, the magnetic Barkhausen noise (MBN) method is a mature non-destructive examination technique for microstructural modifications, observation of surface defects caused by abusive manufacturing processes and residual stress [19,20,21]. MBN has its origins from the B–H hysteresis loop, which is not a smooth curve as the magnetic flux density versus the intensity of the magnetic field results in a curve that is instead described as a non-linear step function. These steps correlate with the irregular fluctuations in the magnetization when energized from cyclic excitation provided by ferrous yokes to excite the material area under interest. These steps or jumps of domains form Barkhausen noise and are provided from magnetic domain motion which is the basis of the Barkhausen signal. Moreover, until the applied field is increased sufficiently, pining sites restricts the moving domain wall. When the magnetizing field is reached, the sudden and discontinuous movement of domain walls result sudden changes in magnetization. In the case of microstructural characteristics “defects” such as dislocations, precipitations and segregations cause pinning of the moving domain walls and promote Barkhausen signal changes [22]. MBN is measured via a pick-up coil (independent to the energizing yoke) in the form of a voltage signal significant of surface eddy currents experienced near the surface of the material.

Magnetic Barkhausen measurements were performed by using a Rollscan 350 MBN analyzer, equipped with a Stresstech general-purpose sensor [23]. The magneto elastic parameter (mp) signifying the root mean square (RMS) value is a function of the magnetizing current, voltage and frequency. Each measurement consisted of periodic bursts of MBN signals for a set duration of ten seconds. MBN RMS can be calculated from such signal bursts. The RMS of the MBN signals is expressed as:

$$\mathrm{RMS}=\sqrt{\frac{{\sum_{i=1}^{n}yi}^{2}}{n}}$$

Here *n* is the total number of MBN signals obtained in the particular frequency range, and *y_i_* is the amplitude of the individual burst.

The main instrumentation input parameters are voltage and frequency, and these are determined from voltage and frequency sweeps giving an optimum value for a specific material under test. In addition, the sinusoidal excitation field can be changed to a triangular one however sinusoidal was considered the optimum waveform for the tests carried out during this work. It should also be noted that the applied field frequency has an influence on the depth where the MBN reading is obtained. The lower the frequency the larger the measured depth. Between 0.01 mm and 1 mm penetration depth was achieved where a band pass filter of between 70 and 200 kHz was selected for channeling the pick-up signals of interest.

Scatter of magnetic output responses vs. DBTT was also found with MBN. It was considered such scatter is due to the material microstructure differences as measurement uncertainty was minimized as much as possible, this is in terms of sensor pick-off, surface quality and applied force. The measurement testing regime used a three times sensor pick-off (physical movement of the sensor but same position test point maintained) followed by 5 measurements each time the sensor touched the surface of the material.

## 3. Results

### 3.1. Results of MAT, 3MA and MBN Measurements Made on All Samples

#### 3.1.1. Evaluation of Data without Normalization

In Figure 3 it is shown how the optimally chosen MAT descriptor depends on the transition temperature for the two investigated materials, 15kH2NMFA and A508 Cl.2. We use the terminology “Optimally chosen MAT descriptor” for those parameters, picked up from the generated big data pool, which characterize the best the correlation with the given independent parameter. In the present case this is the material embrittlement generated by neutron irradiation [8,9]. This parameter ensures the largest sensitivity together with good reproducibility. In the case plotted in Figure 3, this descriptor is characterized by h_a_ = −30 mA and h_b_ = 1080 mA magnetic field values for 15kH2NMFA material and by h_a_ = −780 mA and h_b_ = 1200 mA values for A508 Cl.2. material. (h_a_: magnetizing field, h_b_: minor loop amplitude).

Similarly, the results of 3MA are shown in Figure 4. By applying 3MA, clear trend between several magnetic parameters and DBBT was found. In Figure 4 the P3 parameter is given. This is the amplitude of the third harmonics obtained from upper harmonics analysis in the time domain signal of the magnetizing current. Results of MBN measurements, the RMS parameter as a function of transition temperature for all measured 15kH2NMFA and A508 Cl.2 samples can be seen in Figure 5.

It is seen that irradiation caused salient measurable modification of magnetic parameters. Magnetic parameters are significantly affected by the material degradation that changes the DBTT and there is a more or less linear correlation between magnetic parameters and DBTT (except the MBN measurements performed on A508 Cl.2 samples). However, the most visible conclusion, drawn from all measurements is the big scatter of points, regardless of the actual measurement method.

It can also seen very well in Figure 3, Figure 4 and Figure 5 that even the magnetic parameters of not irradiated (reference) samples scatter a lot. This fact gives a possible reason of scatter of measurements points: the samples behave rather differently, despite the fact that the Charpy specimens were cut from the same block. Magnetic measurements do not make anything else but reflect this material inhomogeneity. It is not a surprise that the points will scatter also after irradiation. To have an impression about the behavior of individual samples, the next section investigates how the magnetic properties of individual samples are modified due to neutron irradiation.

#### 3.1.2. Evaluation of Normalized Data

**Figure 6:**
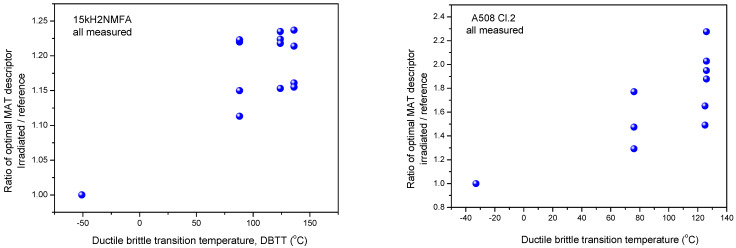
Normalized MAT descriptor vs. transition temperature for all 15kH2NMFA and A508Cl.2 samples.

**Figure 7:**
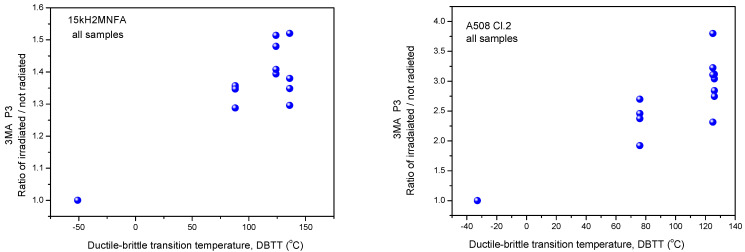
Normalized 3MA P3 parameter as function of transition temperature for all 15kH2NMFA and A508Cl.2 samples.

**Figure 8:**
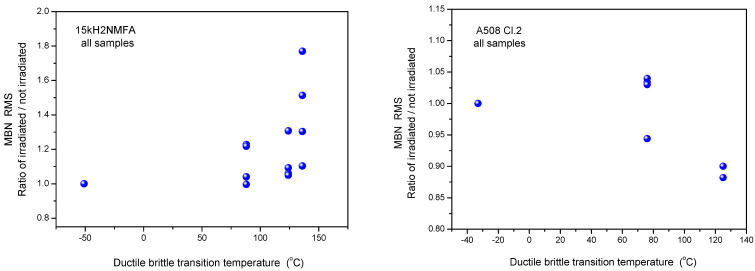
Normalized MBN RMS parameter as function of transition temperature for all 15kH2NMFA and A508Cl.2 samples.

In Figure 3, Figure 4 and Figure 5, all measurement results are given and samples are not marked. Another—and perhaps more useful—way is to consider the change of magnetic parameters for each individual sample. For this purpose, other graphs are shown below (Figure 6, Figure 7 and Figure 8). In these graphs, the modifications of magnetic characteristics are given, this is with respect to the same magnetic parameter that is obtained on the same sample before irradiation giving a baseline condition. This means that the first point (Ratio = 1) is the same for all samples, while each of the other points are connected with specific numbered samples. These points represent how the magnetic behavior of a given sample was modified due to neutron irradiation. (The labeling of points is avoided in order to preserve the clarity of the graphs.)

As can be seen very well in the above graphs, the scatter of points is rather large in the normalized cases, too. This is proof that the scatter of points in Figure 3, Figure 4 and Figure 5 is not the result of the originally different behavior, but also of the fact that neutron irradiation generates different material embrittlement, depending on the individual samples’ behavior.

### 3.2. Selection of Samples

In the above sections, the influence of neutron irradiation has been investigated as if all measured samples are taken into account. As already mentioned above, it has been found that even reference samples are different from the point of view of magnetic properties, so it is not surprising that they behave differently also after irradiation. In this section, the method of the selection of samples is presented, based on permeability measurements of the samples. In the following section, it will be shown how the correlation of magnetic parameters with DBTT looks if only the selected samples are taken into consideration.

The selection of samples is based on measured permeability loops. Evidently this selection was made before any further evaluation of irradiated samples. These permeability loops were measured on reference samples (before irradiation). The criteria in this case was the similarity of the magnetic behavior. These samples were selected which were similar to each other from a magnetic point of view. A good characteristic is the maximal permeability, which can be determined easily from directly measured permeability loops. This means that this selection does not take into account the neutron irradiation generated material embrittlement, it reflects solely on the behavior of samples with initial conditions.

It is emphasized that we did not use backward reasoning to decide which data points fit the best to our hypothesis. Clarifying this statement, the selection process is shown: (1) A large scatter of all magnetic parameters measured on irradiated and reference samples was observed. (2) Independently of the result of magnetic measurements, the magnetic behaviors of the reference samples were compared to each other. Several samples were found with very similar permeability curves. (3) The MAT, 3MA and MBN evaluations were made again, but only the selected, magnetically similar samples were taken into account. No information about the behavior of the irradiated samples was available, since selection was performed prior to irradiation.

Selection reduces only the number of samples, which are taken into account. A serious argument for this selection is, that in the case of the 3MA and MBN method, this selection resulted in a very similar result as in the case of MAT method.

**Figure 9:**
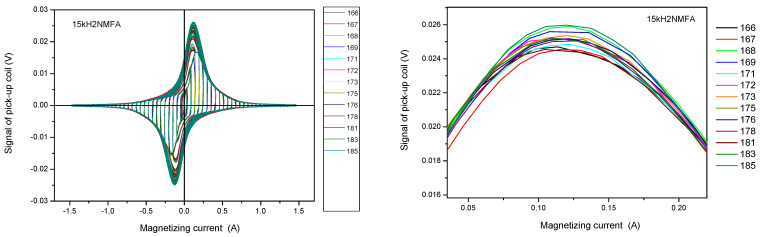
Measured permeability loops of 15kH2NMFA samples before irradiation. The right panel shows the magnified part of the left graph [11].

The series of permeability loops measured on 15kH2NMFA samples are shown in the left side of Figure 9. Magnified parts of the loops can be seen in the right side of the figure, but here only the envelope of the large amplitude minor loops are presented, to make visible the difference between loops, and to provide easy selection from a visual perspective. Four samples have been found that are similar from magnetic point of view. These samples are numbers 172, 173, 178, and 183.

**Figure 10:**
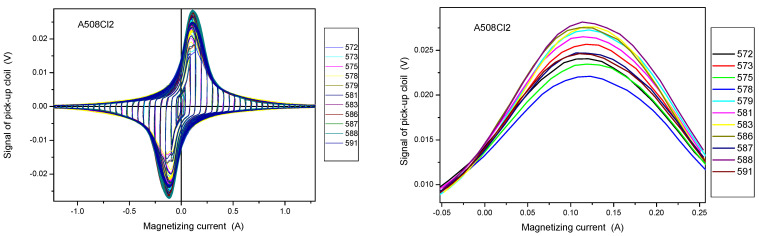
Measured permeability loops of A508 Cl.2 samples before irradiation. The right panel shows the magnified part of the left graph.

Series of permeability loops measured on A508 Cl.2 samples are shown in Figure 10. Again, four samples have been found, which are similar from magnetic point of view. These samples are numbers 579, 583, 586, and 588.

### 3.3. Results of 3MA, MAT and MBN Measurements Considering Selected Samples Only

**Figure 11:**
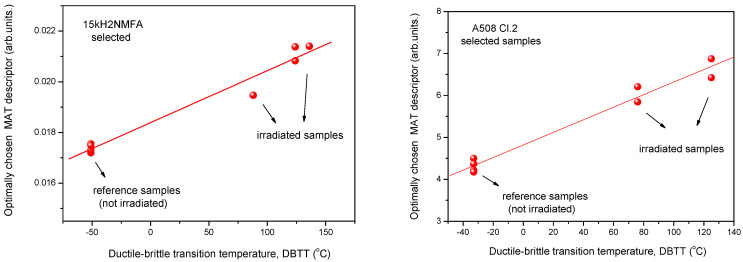
Optimally chosen MAT descriptor vs. transition temperature for selected 15kH2NMFA and A508 Cl.2 samples.

**Figure 12:**
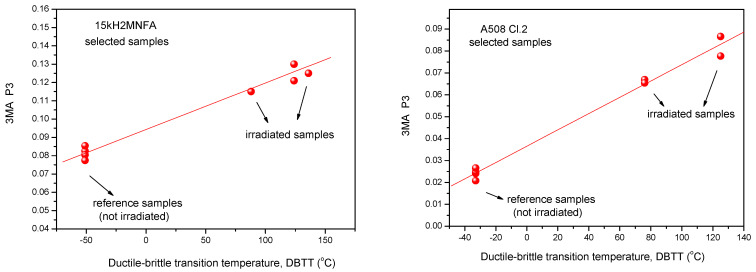
3MA P3 parameter vs. transition temperature for selected 15kH2NMFA and A508 Cl.2 samples.

**Figure 13:**
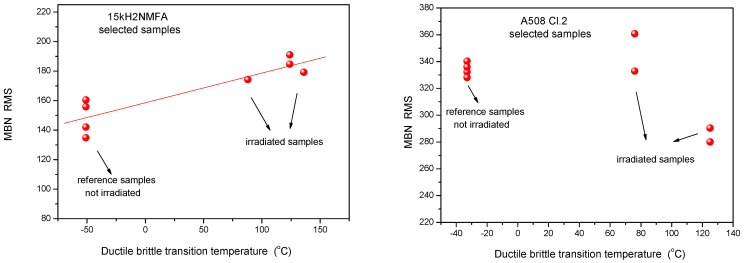
MBN RMS parameter as a function of transition temperature for selected 15kH2NMFA and A508 Cl.2 samples.

In this section it is shown how the scatter of points is modified if the evaluation of magnetic parameters has been repeated taking into account only the magnetically pre-selected samples. Results are shown in Figure 11, Figure 12 and Figure 13, respectively.

## 4. Discussion

By this analysis it has been proven that the experienced big scatter is connected with the different behavior of the samples, and the reason is not really the measurement errors of the applied magnetic methods.

It should be emphasized that MAT descriptors were determined for all the samples independently, before any selection and later any time (see Figure 11) the same parameters (h_a_ = −30 mA, h_b_ = 1080 mA for 15kH2NMFA and h_a_ = 780 mA, h_b_ = 1200 mA for A508 Cl.2) were used. Selection reduces only the number of samples, which are taken into account. A serious argument for this selection is, that in the case of 3MA and MBN method, this selection resulted in a very similar result as that in the case of the MAT method.

If we compare Figure 11 with Figure 3, Figure 12 with Figure 4 and Figure 13 with Figure 5, it can be seen that the scatter of points dramatically decreased if evaluation was performed only on the selected samples with similar magnetic behavior. An obvious linear correlation with low scatter of points has been found between magnetic parameters and DBTT for both investigated materials and for the three considered magnetic methods. One exception is the MBN RMS parameter for the A508 Cl.2 material. In this latter case, the scatter has been also decreased, similarly to all other cases, but we cannot speak about neither linear nor even monotonous correlation. This observation needs some more discussion. However, the correlation between MAT and 3MA measurements are more than satisfactory. Neither the correlation between magnetic parameters and DBTT, nor the behavior of scatter does not depend on the actual method of measurement. This fact is very promising for the future practical application of magnetic methods. The results of the different methods verify one another.

We have found relevant differences in magnetic behavior, which resulted in big scatter in MAT, 3MA and also in MBN vs. DBTT plots. These differences are rather surprising and unexpected, because the samples were cut from the same block. As three different NDT methods indicated the differences, these can not be assigned to the uncertainity of any one of them, although the structure and chemical composition of the different samples should be the same. Describing this effect we cannot use any other word than ”inhomogeneity of the material”, without knowing anything about the character of inhomogeneity. This result is considered one of the most important messages of our work.

In this paper we have presented figures about the scattering of the destructive mechanical tests and of the non-destructive magnetic measurements. Both types of experiments indicate that the source of the observed scattering is related to the differences between the tested specimens either from a mechanical or magnetic point of view. We cannot provide evidence that the scattering of the mechanical properties and of the magnetic features have identical causes. However, the quality of the linear relationship between the determined DBTT and the MAT, 3MA, and MBN values can be considered as a telling argument in this direction.

We know that further analysis to verify the effect of the local inhomogeneity of the material is extremely important and perhaps this result would be crucial for the whole nuclear industry. We believe that if we call the attention of the scientific community to this fact, it is important by itself. Evidently, the work should be continued, and we want to do this.

## 5. Conclusions

Neutron irradiation-generated embrittlement was investigated by three different types of nondestructive magnetic methods on two different types of reactor pressure vessel steel materials and the results were compared with the destructively measured transition temperature. A reasonable correlation was found between magnetic parameters and DBTT, which can be used in future potential applications to estimate DBTT from the results of magnetic measurement. A good correlation was found, as well, between the results obtained by the different methods.

The present work is considered as a direct continuation of Reference [11]. In this, recently published paper, a possible explanation was given for the big scatter of nondestructively measured magnetic characteristics as functions of transition temperature. By applying the so-called MAT method, a possible reason has been found for the scatter. Here, two other principally rather than different magnetic methods have been applied on the same series of samples, and also, on another nuclear pressure vessel steel material having different chemical composition and different properties in order to establish a much larger context of the source of the observed scatter, which was not entirely explained in our previous paper. The results from the other methods were surprisingly similar as in the case of MAT. This means:

- Verification of MAT measurements;
- Proving that the former MAT result is not methodology dependent;
- Proving that the former MAT result is not material dependent.

A common feature of different techniques—large scatter of points—was also analyzed. As an explanation, this scatter was attributed to the local material inhomogeneity. It was shown that the measurement error is not responsible for the scatter. It was clearly demonstrated in these experiments that, if the behavior of the reference (non-irradiated) samples are similar to each other, the irradiation-induced embrittlement can be determined very well, and in this case the scatter of the magnetic parameters is very low.

One of the most important conclusions of this work is, that the parameters determined by magnetic measurements seem to characterize better the neutron generated material embrittlement than the conventionally used destructive methods. The scatter of the magnetic results is lower than the scatter of the Charpy tests. In addition, the magnetic method characterized the actual, individual samples, in contrary to the transition temperature values determined by the Charpy impact testing methods, which can provide only statistical values on the set of samples.

Another important conclusion is that local material inhomogeneities have a great influence on the neutron irradiation-induced material embrittlement. Different parts of the reactor pressure vessel, even if they are cut from the same larger block, are hardened differently. Taking into account the measurement conditions’ analysis, local material conditions can be responsible for the different neutron irradiation generated embrittlement of the pressure vessel steel material caused by the same dosage of neutron irradiation.

These facts mean a telling argument for the application of non-destructive magnetic measurements in the reactor industry for future operations.

## Figures and Tables

**Table 1:** 15Kh2NMFA base metal chemical composition (wt %) of the.

C	Si	Mn	Cr	S	P	Ni	Mo	Cu	V
0.16	0.29	0.42	1.97	0.008	0.012	1.29	0.52	0.12	0.12

**Table 2:** A508 Cl.2 base metal chemical composition (wt %) of the.

C	Si	Mn	Cr	S	P	Ni	Mo	Cu
0.201	0.27	0.578	0.372	0.0085	0.0091	0.668	0.599	0.0472

**Table 3:** Fast fluence (E > 1 MeV) and DBTT for A508 Cl.2 material.

Fast Fluence (E > 1 MeV) (×10^19^ n/cm^2^)	DBTT T_41J_ (°C)
0	−33 ± 9
1.55	76 ± 15
4.38	125 ± 15
7.04	126 ± 15

**Table 4:** Fast fluence (E > 1MeV) and DBTT for 15Kh2NMFA material.

Fast Fluence (E > 1 MeV) (×10^19^ n/cm^2^)	DBTT T_41J_ (°C)
0	−51 ± 12
2.78	88 ± 15
6.83	136 ± 15
7.9	124 ± 15

## Data Availability

The data are contained within the article.
